# Brief Comparison of Novel Influenza Vaccine Design Strategies

**DOI:** 10.3390/vaccines13111164

**Published:** 2025-11-15

**Authors:** Shiqi Chai, Chuantao Ye, Chao Fan, Hong Jiang

**Affiliations:** Department of Infectious Diseases, Tangdu Hospital of the Fourth Military Medical University, Xi’an 710038, China; 18820134730@163.com (S.C.); yechuantao2008@163.com (C.Y.)

**Keywords:** vaccine, influenza, virus, design strategies, delivery platform

## Abstract

Influenza viruses remain a major global public health concern, causing significant morbidity and mortality annually despite widespread vaccination efforts. The limitations of current seasonal vaccines, including strain-specific efficacy and manufacturing delays, have accelerated the development of next-generation candidates aiming for universal protection. This review comprehensively summarizes the recent progress in universal influenza vaccine research. We first outline the key conserved antigenic targets, such as the hemagglutinin (HA) stem, neuraminidase (NA), and matrix proteins (M2e, NP, and M1), which are crucial for eliciting broad cross-reactive immunity. We then delve into advanced antigen design strategies, including immunofocusing, multi-antigen combinations, computationally optimized broadly reactive antigens (COBRA), and nanoparticle-based platforms. Furthermore, we evaluate evolving vaccine delivery systems, from traditional inactivated and live-attenuated vaccines to modern mRNA and viral vector platforms, alongside the critical role of novel adjuvants in enhancing immune responses. The convergence of these disciplines—structural biology, computational design, and nanotechnology—is driving the field toward a transformative goal. We conclude that the successful development of a universal influenza vaccine will likely depend on the strategic integration of these innovative approaches to overcome existing immunological and logistical challenges, ultimately providing durable and broad-spectrum protection against diverse influenza virus strains.

## 1. Introduction

Influenza, an acute respiratory infection caused by influenza viruses, remains a persistent global public health threat due to its ability to cause seasonal epidemics and occasional pandemics [1]. Characterized by high morbidity and transmissibility, the disease spreads primarily through respiratory droplets from infected individuals, as well as via contact with contaminated surfaces [2]. Seasonal influenza outbreaks result in an estimated 1 billion cases each year worldwide, including 3 to 5 million severe cases and 290,000 to 650,000 respiratory-related deaths [3,4]. The burden of influenza mortality varies by region and age group: in industrialized nations, most deaths occur among those aged 65 and older [5], whereas 99% of deaths in children under 5 years of age with influenza-related lower respiratory tract infections are found in developing countries [6]. Beyond its health impact, influenza contributes to significant socioeconomic losses through healthcare spending, worker absenteeism and long-term human-capital loss.

Vaccination represents the most effective strategy for influenza prevention. Seasonal influenza vaccines are designed to protect against the virus strains predicted to circulate in a given season [7]. For the 2024–2025 influenza season in the United States, trivalent formulations—including inactivated (inactivated influenza vaccine, Trivalent, IIV3), recombinant (recombinant influenza vaccine, Trivalent, RIV3), and live-attenuated (live-attenuated influenza vaccine, Trivalent, LAIV3) vaccines—are expected to be available [8]. Vaccine composition is updated annually based on global surveillance data and may include strains from the H1N1, H3N2, and B/Victoria lineages, depending on the manufacturing method [9].

Despite their widespread use, current influenza vaccines face several limitations. Most seasonal and pandemic influenza vaccines are still produced using egg-based systems, which account for the majority of the inactivated influenza vaccine supply [10]. This production method has notable drawbacks, including vulnerability to supply chain disruptions due to dependence on embryonated eggs, and the potential for vaccine strain mutations during egg adaptation, which can impair effectiveness [11]. Additionally, due to antigenic drift in circulating viruses, vaccine efficacy can vary widely, ranging from 20% to 60% in some seasons [12].

In response to these challenges, efforts are intensifying to develop next-generation influenza vaccines. mRNA-based platforms represent a highly promising alternative [13]. These vaccines use lipid nanoparticle-encapsulated mRNA encoding specific viral antigens to elicit robust humoral and cellular immune responses [14]. Another innovative strategy involves proteolysis-targeting (PROTAR) vaccines, which leverage the host ubiquitin–proteasome system to conditionally regulate viral protein stability and degradation [15]. Clinical studies indicate favorable safety, immunogenicity, and cross-protective potential for such candidates [16].

In summary, despite ongoing vaccination efforts, there remains a critical need for more effective, efficient, and adaptable influenza vaccines. A thorough understanding of influenza virology and epidemiology, combined with insights into current vaccine strengths and limitations, is essential to guide the development of next-generation vaccines capable of providing broader and more durable protection for global public health.

## 2. Antigenic Targets of Universal Influenza Vaccines

**Figure 1:**
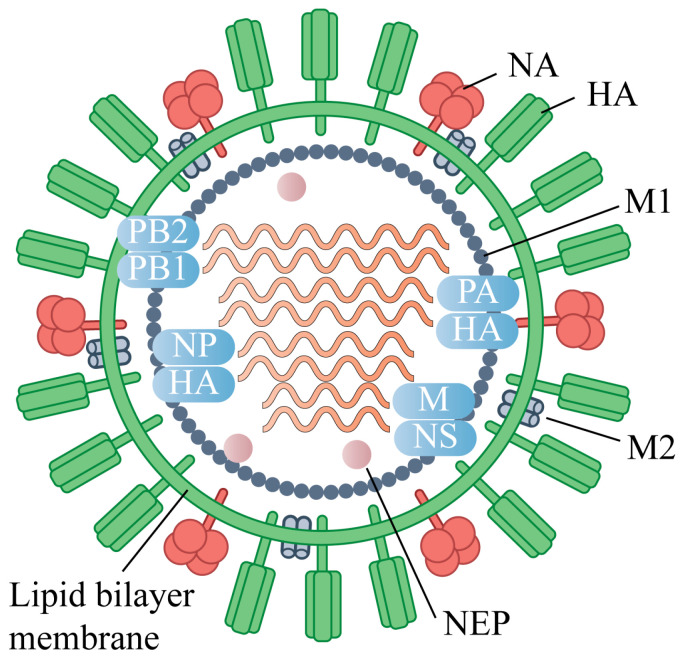
Virion Structure of the Influenza A virus (IAV).The IAV virion consists of a lipid envelope that contains the glycoproteins hemagglutinin (HA), neuraminidase (NA), and the matrix protein 2 (M2) ion channel. Below the envelope is a layer of matrix protein 1 (M1) and nuclear export protein (NEP). The IAV genome is composed of eight segmented vRNAs: polymerase basic protein 2 (PB2), polymerase basic protein 1 (PB1), polymerase acidic protein (PA), HA, nucleoprotein (NP), NA, M, and non-structural protein (NS).

Influenza viruses belong to the *Orthomy xoviridae* family and possess a genome consisting of eight segments of single-stranded negative-sense RNA, encoding at least 11 viral proteins. Based on antigenic differences in nucleoprotein (NP) and matrix protein (M), influenza viruses are classified into four types: A, B, C, and D [17] (Figure 1). Among these, influenza A and B viruses are the main causative agents of seasonal influenza in humans. Influenza A viruses are further subdivided into 18 hemagglutinin (HA) and 11 neuraminidase (NA) subtypes according to their surface glycoproteins [18]. Subtypes H1–H3 and N1–N2 represent the predominant seasonal strains, while avian-origin subtypes such as H5, H7, and H9 have repeatedly crossed species barriers to infect humans [19], often causing severe disease with high mortality and posing ongoing threats to global public health.

HA, NA, NP, M1, and M2 represent the principal antigenic targets under investigation for the development of universal influenza vaccines. The HA protein facilitates viral entry by binding to sialic acid receptors and mediating membrane fusion within endosomes [20]. Structurally, HA forms a homotrimer, with each monomer comprising a globular head domain and a stem region. The globular head is immunodominant and the primary target of strain-specific neutralizing antibodies. However, due to antigenic drift, such antibodies often exhibit limited cross-reactivity [21,22]. In contrast, the stem region of HA is highly conserved and harbors multiple B- and T-cell epitopes [23]. Antibodies targeting the HA stem have demonstrated broad neutralizing activity across influenza subtypes [24,25], highlighting its promise as a universal vaccine candidate. For instance, an equine H3N8 HA-based immunogen recently elicited antibodies reactive against multiple human group 1 and 2 viruses [26], underscoring the potential for cross-species protection. Historically, stem-directed antibody responses have been difficult to elicit with conventional vaccine platforms, often requiring stabilized trimeric immunogens and repeated immunizations to achieve robustness [27]. Overcoming this immune subdominance remains a central challenge in stem-based universal vaccine design. Novel strategies like mRNA delivery are challenging this paradigm.

NA is a tetrameric type II transmembrane glycoprotein that cleaves sialic acid residues from mucins, facilitating viral mobility, release of progeny virions, and prevention of self-aggregation [28]. Antibodies against NA can confer protection by inhibiting viral release or mediating antibody-dependent cellular cytotoxicity (ADCC). The enzymatic active site of NA (residues 222–230) is highly conserved across influenza A and B viruses [29], and anti-NA antibodies have demonstrated both homotypic and heterosubtypic protection [30,31,32]. However, NA is generally less immunogenic than HA and lacks immunodominance in conventional vaccines [28]. Notably, when mice were vaccinated twice at a 3-week interval intranasally or intramuscularly, mucosal NA vaccination has been shown to induce superior protection compared to intramuscular administration, maybe due to mucosal antibodies including secretory IgA [30]. Furthermore, broad-inhibition NA-targeting memory B cells are present in healthy adults, representing a plausible target for next-generation vaccines [33]. Recent advances include the AI-assisted design of genetic algorithm-based mosaic NA antigens aimed at broadening immune coverage.

The M2 protein is a tetrameric proton channel embedded in the viral envelope that facilitates viral uncoating by acidifying the virion interior [34,35]. Its N-terminal extracellular domain (M2e) is highly conserved—comprising only 23 amino acids—and is unlikely to accumulate escape mutations [36,37]. Although M2e is poorly immunogenic during natural infection or conventional vaccination, M2e-specific antibodies can mediate ADCC or complement-dependent cytolysis (CDC) of infected cells [38,39]. As a short peptide, M2e alone suffers from poor stability and weak immunogenicity [36], necessitating fusion to carrier platforms or incorporation into multivalent nanoparticle vaccines to enhance immunogenicity. For example, a tandem antigen nanoparticle displaying three M2e domains from influenza A (human and avian/swine strains) and three from influenza B conferred 100% protection against lethal H1N1 challenge and 70% protection against influenza B in mice [40]. Such carrier-fusion strategies represent a viable path for leveraging M2e in universal vaccine design [41,42].

NP and M1 are major internal structural proteins of the influenza virus: M1 underpins viral assembly, while NP binds viral RNA to form ribonucleoprotein complexes essential for replication [43]. Both are relatively conserved and contain epitopes recognized by cross-reactive cytotoxic T lymphocytes (CTLs) [44,45,46]. Although CTL responses can reduce disease severity and mortality, they do not prevent infection and often require synergistic humoral immunity for full protection [47]. Therefore, NP and M1 are often incorporated into multi-antigen vaccine regimens. In a Phase 2a controlled trial involving healthy adults aged 18–55, a candidate vaccine displaying seven copies of NP induced robust humoral and cellular immunity and showed preliminary efficacy (VE = 84%) against influenza following a single intramuscular dose [48], supporting the potential of internal proteins in universal vaccine strategies.

## 3. Antigen Design Strategies for Universal Influenza Vaccines

The pursuit of a universal influenza vaccine centers on targeting conserved viral regions to elicit broad and durable protection. Key design strategies currently under investigation include immunofocusing, multi-antigen combinations, computationally optimized broadly reactive antigens (COBRA), and nanoparticle-based antigen presentation.

### 3.1. Immunofocusing Strategy

Immunofocusing aims to direct immune responses toward conserved, functionally critical epitopes while minimizing distraction by variable immunodominant regions. A prominent example involves the use of chimeric hemagglutinin (HA) constructs, where animals immunized with HAs bearing the same conserved stalk domain but different, irrelevant head domains develop potent stalk-directed antibody responses [49]. Another approach leveraged an avian H5N1 HA backbone by grafting a human influenza HA head domain, successfully inducing broadly cross-reactive antibodies targeting the HA stem in preclinical models [50]. Early concepts of head-domain removal to unmask conserved regions date back to the 1980s [51], and recent advances include vaccine nanoparticles displaying tandem copies of an α-helical stem fragment, which conferred heterosubtypic protection in mice [52]. Alternatively, conformational masking of the HA head using aluminum adjuvant attachment has been shown to redirect antibody production toward the stem region, broadening reactivity across subtypes [53]. A central challenge in immunofocusing, however, lies in preserving the native conformation of target epitopes during antigen design—a particular hurdle for structurally complex proteins [50].

### 3.2. Multi-Antigen Combination Strategy

Integrating multiple antigens into a single formulation represents a promising approach to broaden immune coverage. Covalent coupling of HAs from different strains has been shown to enhance breadth of response and reduce biases linked to “original antigenic sin” [54]. Structure-guided chimeric immunogens—such as grafting an H3 receptor-binding site onto an H1 HA scaffold—have elicited neutralizing antibodies against diverse strains [55]. Nucleic acid platforms also support this strategy; for example, an mRNA construct co-expressing HA and NA induced cross-reactive antibodies against heterologous viruses [56]. Fusions of conserved internal and exterior proteins, such as M2e and nucleoprotein, delivered with lipid nanoadjuvants, have provided protection against homologous and heterosubtypic challenges [57]. Multivalent epitope-based nanoparticles are under active investigation [58,59], including virus-like particles displaying M2e from human and avian influenza A viruses, which broke host restriction and conferred complete cross-protection in mice [60]. Despite these advances, multi-antigen vaccines face challenges such as imbalanced immunity toward specific epitopes, increased antigen load raising safety concerns, and difficulties in maintaining the native conformation of each antigen within a complex formulation.

### 3.3. COBRA Strategy

The COBRA approach leverages bioinformatics to design immunogens based on layered consensus sequences from viral databases, aiming to maximize coverage of past, present, and potential future strains. COBRAs are generated through iterative sequence alignment and consensus-building steps, yielding synthetic proteins with enhanced cross-reactive potential. A COBRA vaccine targeting the H1 HA head was first reported in 2016 and shown to elicit broad antibodies against drifted H1N1 strains [61]. Similarly, H3-focused COBRA HA immunogens outperformed wild-type HA vaccines in breadth and potency against historical and emerging variants [62,63], including in studies involving elderly populations [63]. Extension of COBRA to neuraminidase (NA) has yielded candidates that induce wider cross-reactive antibody responses than wild-type NA [64]. Adjuvants such as cyclic GMP-AMP (STING agonist), AddaVax, CpG, and Alhydrogel have been tested in combination with COBRA immunogens to further enhance immunogenicity [65,66]. A limitation of early COBRA designs is their dependence on pre-existing viral sequences, which may not anticipate novel reassortants. Next-generation COBRA strategies are now being developed to improve predictive coverage and responsiveness to emerging strains.

### 3.4. Nanoparticle

Nanoparticle-based vaccines enable high-density, ordered display of influenza antigens, improving immunogenicity and often incorporating self-adjuvanting properties. Their small size (20–200 nm) facilitates efficient drainage to lymph nodes, enhancing antigen presentation. For example, ferritin nanoparticles displaying conserved epitopes from the HA stem, NP, and M2e induced durable cross-reactive immunity lasting at least six months post-vaccination [58]. Similarly, Epigraph-designed H9 HA trimers conjugated to mi3 nanoparticles elicited broad antibody and T-cell responses, conferring superior protection against diverse H9N2 viruses [67]. A baculovirus-derived nanoparticle co-expressing HA, triple-repeat M2e, and an M-cell-targeting ligand provided comprehensive protection across multiple influenza A subtypes [68]. Other platforms, including self-adjuvanting PLGA, lipid-polymer hybrids, and hydrogel nanoparticles, have also been shown to induce broad and persistent immune responses [57,69,70]. Despite their promise, nanoparticle vaccines face challenges in reproducible size control [71], with evidence suggesting that larger particles may be more efficiently internalized by antigen-presenting cells and promote stronger T-cell activation [72]. Antigen valency and spatial arrangement are also critical; although high density can enhance B-cell activation, overly dense epitope presentation may sterically hinder receptor binding, necessitating careful optimization [73].

Future universal influenza vaccine design will likely integrate the strengths of multiple platforms—combining rational epitope selection, structural vaccinology, computational forecasting, and advanced delivery systems. A streamlined, multi-target approach emphasizing conserved regions and balanced humoral and cellular immunity will be essential to achieve broad, durable, and effective protection against diverse influenza viruses.

## 4. Vaccine Delivery Platform

Vaccination is an important way to achieve herd immunity, which plays an important role in the control of COVID-19. For the influenza virus, making the immunogens available to establish herd immunity is also important. To achieve this goal, in addition to improving vaccination coverage, it is also essential to follow the principle of prioritizing the protection of high-risk populations. Therefore, the development of vaccines for populations including the elderly, children, pregnant women, and patients with chronic disease is crucial. Meanwhile, the tendency of the influenza virus to mutate also hinders the establishment of herd immunity. The platform should also meet the needs of multiple responses to mutant strains.

Conventional influenza vaccine platforms—including inactivated vaccines, live attenuated vaccines, and recombinant protein-based vaccines—have long served as the cornerstone of influenza prophylaxis, providing established safety and stability profiles. Influenza vaccines administered via the nasal route have demonstrated great potential in influenza prevention by inducing strong local and systemic immune responses [58,68,74]. However, these traditional systems face challenges in rapidly addressing the continuous emergence of new viral strains.

Next-generation delivery platforms offer improved adaptability, manufacturing speed, and immunogenicity, positioning them as promising candidates for future influenza vaccines, especially in pandemic settings. Key emerging technologies include recombinant viral vector vaccines, conjugate vaccines, and nucleic acid-based vaccines. The application of nucleic acid-based vaccines, such as mRNA vaccines, during the SARS-CoV-2 pandemic has enriched the evidence of side effects associated with this kind of vaccine, including swelling at the injection site, fatigue, headache, muscle soreness, fever, etc. Serious side effects include myocarditis and allergic reactions. Therefore, the improvement should be made before their widespread application.

The traditional and new vaccines under development were summarized below (Table 1).

### 4.1. Traditional Vaccines Under Development

#### 4.1.1. Inactivated Vaccines

Traditional inactivated influenza vaccines are produced by propagating the influenza virus in embryonated chicken eggs, followed by chemical inactivation. Although cost-effective, egg-based production is associated with egg-adaptive mutations that may reduce vaccine effectiveness, and reactogenicity such as fever has been reported. Advances in purification, adjuvantation, and the adoption of cell culture-based production systems have improved the safety and immunogenicity of these vaccines [75].

Further refinements have led to split-virion and subunit vaccines, which are less reactogenic and offer improved safety profiles. Split vaccines are generated by detergent disruption of whole virions, whereas subunit vaccines are highly purified formulations containing mainly hemagglutinin (HA) and neuraminidase (NA) [76]. Although the standard production timeline for seasonal influenza vaccines remains around six months, cell culture systems are being increasingly implemented to shorten production time and avoid egg-dependent limitations. Nevertheless, achieving high yield and cost-effectiveness in cell-based systems remains a challenge [75].

#### 4.1.2. Live Attenuated Vaccine

Live attenuated influenza vaccines (LAIVs) are generated by reducing viral virulence through methods such as cold adaptation, while retaining the ability to replicate to a limited extent in the respiratory tract. These vaccines induce both humoral and cellular immunity, including mucosal IgA and systemic IgG responses, and have been associated with favorable T-cell responses that confer heterologous protection [77,78]. However, due to the potential for vaccine-related adverse events, LAIVs are generally not recommended for certain populations, including immunocompromised individuals, pregnant women, and the elderly [75].

Novel attenuation strategies are under investigation to improve safety profiles. For example, proteolysis-targeting chimeric (PROTAC) technology has been employed to generate conditionally destabilized influenza viruses, enabling controlled attenuation and enhancing vaccine safety in preclinical studies [79,80].

#### 4.1.3. Recombinant Protein-Based Vaccine

Recombinant protein vaccines utilize purified viral proteins or epitopes as immunogens, offering a favorable safety profile compared to whole-virus or live attenuated platforms. Their design relies on prior knowledge of antigenic epitopes and genetic information, which can prolong development timelines. However, for influenza, well-established targets such as HA, NA, and M2e allow for flexible and rapid antigen design. HA-based recombinant vaccines can be produced in mammalian or insect cell systems within approximately two months, facilitating a rapid response to emerging strains [81].

Innovations in antigen design include mosaic HA (mHA) constructs that integrate conserved epitopes from multiple strains to broaden immune coverage [82]. Additionally, computationally optimized broadly reactive antigen (COBRA) methodologies, increasingly supported by artificial intelligence (AI), are being used to design HA antigens capable of eliciting cross-reactive immunity against diverse influenza variants [65,74,83,84].

**Table 1:** The traditional and new vaccines under development for influenza vaccines.

Types of Vaccines	Characteristic and Optimization	Delivery Methods	Immune Responses	Advantages	Disadvantages	Application Stage
Inactivated Vaccines [74,75]	egg-based production	intramuscular injection	humoral and cellular immunity	cost-effective	egg-adaptive mutations	clinically approved
	cell culture-based production			improved safety and immunogenicity	costly	
	split-virion					
	viral subunit					
Live Attenuated Vaccine [76,77,78,79]	cold adaptation	intranasal administration	humoral and cellular immunity	Natural immune responses	vaccine-related adverse events	clinically approved
	PROTAC			improve safety	enhancing vaccine safety	preclinical studies
Recombinant Protein-Based Vaccine [80,81,82]	purified viral proteins or epitopes as immunogens	intramuscular injection	enhanced humoral immunity	safety/flexible antigen design	the design relies on prior knowledge of the virus	clinically approved
	mosaic HA (mHA)			multiple strains coverage		preclinical studies
	COBRA methodologies			AI-based automation		preclinical studies
Recombinant Viral Vector Vaccines [74,85]	Adenovirus vector	intramuscular/intranasal	TLR-dependent and independent signaling pathways	target specific immune cells	safety concern	preclinical studies
	MVA vector					
	NDV vector					
Conjugate Vaccine [86,87,88]	links poorly immunogenic antigens to carrier proteins	intramuscular/intradermal	T-cell-dependent immunity and memory B-cell formation	improved immune responses	costly	preclinical studies
Nucleic Acid-Based Vaccines	DNA-based [89]	intramuscular/Subcutaneous/intradermal/others	both humoral and cellular immunity	rapid, scalable, and cost-effective	safety concern	preclinical studies
	mRNA-based [89,90,91,92,93,94]					Phase-III-clinical-trial
	saRNA-based [95]					preclinical studies

PROTAC: proteolysis-targeting chimeric technology; COBRA: computationally optimized broadly reactive antigen; MVA: modified vaccinia Ankara; NDV: Newcastle disease virus; AI: artificial intelligence.

### 4.2. Recent Vaccine Platform in Progress

Novel vaccine technologies have been addressed for meeting the needs of the development of efficient, safe, and stable vaccines. The next generation of the vaccine platform should respond quickly to the development and manufacture of the vaccine after an epidemic outbreak. For the influenza vaccine, the vaccine also needs to respond quickly against the emerging variant of influenza. To address these challenges, recombinant viral vector vaccines, conjugate vaccines, and NA-based vaccines have been widely studied. Although some of them have not yet reached the application stage, they could potentially induce an appropriate and safe immune response with an efficient delivery system.

#### 4.2.1. Recombinant Viral Vector Vaccines

Recombinant viral vector vaccines employ engineered, replication-deficient or attenuated viruses to deliver influenza antigens. These vectors often possess intrinsic adjuvant properties and can be designed to target specific immune cells, activating both TLR-dependent and independent signaling pathways. The licensure of adenovirus-vectored COVID-19 vaccines (e.g., Vaxzevria^®^) has validated this platform for respiratory viruses [85]. For influenza, various vectors—including adenovirus, modified vaccinia Ankara (MVA), and Newcastle disease virus—have been engineered to express antigens such as HA, NP, and M1, eliciting robust T-cell responses in preclinical and clinical studies [75].

#### 4.2.2. Conjugate Vaccine

Conjugate vaccine technology, widely used against bacterial pathogens, links poorly immunogenic antigens to carrier proteins to enhance immunogenicity. This approach has recently been applied to viral targets; for example, SARS-CoV-2 antigens conjugated to designer peptides have shown improved immune responses [86,87]. Conjugate vaccines promote T-cell-dependent immunity and memory B-cell formation [79]. In the context of influenza, conjugating low-immunogenicity antigens such as NA or M2e to immunogenic carriers can circumvent the limitations of viral antigenic variation. Moreover, mosaic protein designs enable efficient production in bacterial expression systems, supporting scalable manufacturing [88].

#### 4.2.3. Nucleic Acid-Based Vaccines

Nucleic acid (NA) vaccines comprise DNA or mRNA encoding target antigens and represent a rapid, scalable, and cost-effective vaccine modality. DNA vaccines introduce plasmid DNA into host cells, enabling in situ antigen expression without the need for live virus culture. Although several influenza DNA vaccines have entered early-stage clinical trials, concerns regarding potential genomic integration have limited their widespread adoption [89].

mRNA vaccines have gained prominence as a safe and efficient alternative, with no risk of genomic integration. They elicit both humoral and cellular immunity, with antigen presentation via MHC-I stimulating CD8+ T-cell responses [89,90]. Preclinical studies of mRNA vaccines encoding multiple HA antigens have demonstrated potent immunity and protection in animal models [56,91]. Universal influenza mRNA vaccine strategies include targeting conserved internal proteins (e.g., NP, M1, PB1) or combining multiple antigens from different subtypes (e.g., HA, NA, M2) to achieve broad cross-protection [92,93]. A multivalent mRNA vaccine encoding 20 HA subtypes recently induced broad, subtype-specific antibodies and mitigated influenza symptoms in preclinical models [94].

Self-amplifying RNA (saRNA) vaccines represent a further evolution, encoding a viral replicase that enables intracellular RNA amplification and prolonged antigen expression at lower doses. While saRNA platforms have shown promise for influenza and other pathogens, their safety profiles require further evaluation [95].

A critical factor for NA vaccine efficacy is the delivery system (Table 2). Lipid nanoparticles (LNPs)—composed of ionizable lipids, cholesterol, PEG-lipids, and phospholipids—have been widely adopted for their ability to protect nucleic acids and enhance cellular uptake. Alternative delivery systems under investigation include lipid-polymer hybrids, exosomes, peptide-based carriers, and inorganic nanoparticles [96].

### 4.3. Adjuvant Systems

Adjuvants are incorporated into vaccine formulations to enhance the magnitude, breadth, and durability of immune responses. Currently licensed adjuvants for influenza vaccines include aluminum salts, emulsions such as MF59 and AS03, and Toll-like receptor (TLR) agonists. These adjuvants, consisting of their active ingredient, were summarized accordingly (Table 3).

#### 4.3.1. Aluminum Adjuvants

Aluminum-based adjuvants (e.g., aluminum hydroxide and aluminum phosphate) are among the most widely used in global vaccination programs. They function by forming antigen depots, promoting phagocytosis, and enhancing Th2-biased humoral immunity. However, they elicit relatively weak cellular immune responses and may cause local reactions, limiting their utility in some contexts [97,98].

#### 4.3.2. Emulsion Adjuvants

MF59, an oil-in-water emulsion containing squalene, polysorbate 80, and sorbitan trioleate, enhances antigen uptake and promotes a mixed Th1/Th2 response via MyD88-dependent signaling [99]. Similarly, AS03—composed of squalene, polysorbate 80, and DL-α-tocopherol—strengthens antibody responses and has been used in pandemic influenza vaccines such as Pandemrix™ [100,101].

#### 4.3.3. TLR-Agonists-Based Adjuvants

TLR agonists activate innate immunity and promote robust adaptive responses. Monophosphoryl lipid A (MPLA), a TLR4 agonist, is included in several licensed vaccine formulations (e.g., AS01 in Shingrix^®^ and AS04 in Fendrix^®^) and promotes Th1-skewed immunity [102,103,104,105,106]. Other TLR agonists in clinical use include the TLR7/8 agonist imidazoquinoline, which is used in COVAXIN^®^ and the TLR9 agonist CpG 1018 in Heplisav-B^®^ [107,108,109].

#### 4.3.4. Development of Novel Adjuvants

Emerging adjuvants under investigation include mannan-based compounds, which enhance antigen presentation and have shown promise in allergen immunotherapy trials [110,111]. New TLR agonists in development for influenza include TLR3 agonists (e.g., poly(I:C12U)), TLR5 agonists (e.g., flagellin in VAX128), and imidazoquinolines(TLR7/8 agonists), which have demonstrated potent adjuvant activity in preclinical and clinical studies [103,107,112].

**Table 3:** Adjuvant systems used in influenza vaccines.

Types of Adjuvant	Main Components	Immune Enhancement	Application Stage
Aluminum Adjuvants [97,98]	aluminum hydroxide	Th2-biased humoral immunity	clinically employed
	aluminum phosphate		
Emulsion Adjuvants [100,101]	MF59	mixed Th1/Th2 response	clinically employed
	AS03	strengthens antibody responses	
TLR-Agonists-Based Adjuvants	TLR4 agonist (MPLA) [102,103,104,105,106]	promote innate and robust adaptive responses	clinically employed
	TLR7/8 agonist (imidazoquinoline) [107,108,109]		clinically employed
	TLR9 agonist (CpG 1018) [107,112]		clinically employed
	TLR3 agonists (poly(I:C12U)) [107]		preclinical studies
	TLR5 agonists (flagellin) [103]		preclinical studies
	TLR7/8 agonists (imidazoquinolines) [111,112]		preclinical studies

## 5. Conclusions and Perspective

Despite decades of progress, influenza remains a formidable global health challenge. Current seasonal vaccines, while instrumental in reducing morbidity and mortality, are constrained by their limited breadth of protection and the logistical challenges of annual reformulation. The pursuit of a universal influenza vaccine has thus emerged as a critical scientific priority, driving innovation across multiple fronts.

Substantial advances have been made in identifying conserved antigenic targets, rational antigen design, and novel delivery platforms. Strategies such as immunofocusing on the HA stem, multi-antigen combinations, and computationally optimized antigens (COBRA) are yielding promising candidates with broader reactivity. Concurrently, mRNA and nanoparticle platforms have demonstrated an unprecedented capacity for rapid, scalable production and potent immunogenicity.

Looking forward, the successful development of a universal vaccine will likely depend on the intelligent integration of these strategies. No single approach may suffice; instead, a combination of conserved epitopes, structural vaccinology, and advanced delivery systems will be essential. Key challenges remain, including overcoming immune subdominance of conserved regions, ensuring long-lasting immunity, and establishing robust correlates of protection. Furthermore, the transition of these promising platforms from the laboratory to clinical use will necessitate exhaustive clinical trials to thoroughly demonstrate their effectiveness and safety profiles. Global collaboration and leveraging lessons from COVID-19 vaccine development will be paramount. The ultimate goal is a vaccine that confers durable, broad-spectrum protection against seasonal and pandemic strains, transforming our ability to manage influenza and safeguard public health worldwide.

## Figures and Tables

**Table 2:** Different methods for the delivery of Nucleic Acids in vaccine development [96].

Methods	Mechanism	Characteristic	Applications
Lipid Nanoparticles	cationic lipids facilitate transfection; cholesterol/PEG-lipids enhance the stability	high efficiency for mRNA delivery	clinically approved
Lipid-polymer Hybrids	the combination of polymers and lipids; complementarity in physical stability and biocompatibility	the special properties of polymers are utilized to enhance the effectiveness and targeting of delivery	preclinical studies
Exosomes	natural secretory structures; intercellular communication carriers.	low immunogenicity; ability to penetrate biological barriers; inherent targeting	Phase-I-clinical-trial
Peptide-based Carriers	short peptides spontaneously assemble into nano-fiber or nano-particle structures	biodegradability; programmable molecular	preclinical studies
Inorganic Nanoparticles	inorganic materials (such as gold, silica, and iron oxide) load nucleic acids through physical adsorption or chemical coupling	unique physical properties facilitate the integration of diagnosis and treatment	preclinical studies

## Data Availability

Not applicable.

## Abbreviations

The following abbreviations are used in this manuscript:

ADCC	Antibody-dependent cellular cytotoxicity
AI	Artificial intelligence
CDC	Complement-dependent cytolysis
COBRA	Computationally optimized broadly reactive antigen
CTLs	Cytotoxic T lymphocytes
HA	Hemagglutinin
IAVs	Influenza A viruses
IIV3	Inactivated influenza vaccines
LAIV3	Live-attenuated influenza vaccine
LNPs	Lipid nanoparticles
M1	Matrix protein
M2e	M2 extracellular domain
MHC	Major histocompatibility complex
MPLA	Monophosphoryl lipid A
MVA	Modified Ankara
NP	Nucleoprotein
PB1	Polymerase basic protein 1
PEG	Polyethylene glycol
PLGA	Poly lactic-co-glycolic acid
PROTAC	Proteolysis-targeting chimeric
PROTAR	Proteolysis-targeting
RIV3	Recombinant influenza vaccine
saRNA	Self-amplifying RNA
TLR	Toll-like receptor
vRNA	Viral RNA
